# A Framework for Coverage Path Planning Optimization Based on Point Cloud for Structural Inspection

**DOI:** 10.3390/s21020570

**Published:** 2021-01-15

**Authors:** Iago Z. Biundini, Milena F. Pinto, Aurelio G. Melo, Andre L. M. Marcato, Leonardo M. Honório, Maria J. R. Aguiar

**Affiliations:** 1 Department of Electrical Engineering, Federal University of Juiz de Fora, Juiz de Fora 36036-900, Brazil; iago.biundini@engenharia.ufjf.br (I.Z.B.); aurelio.melo@engenharia.ufjf.br (A.G.M.); leonardo.honorio@ufjf.edu.br (L.M.H.); maria.aguiar@engenharia.ufjf.br (M.J.R.A.); 2 Department of Electronics Engineering, Federal Center for Technological Education of Rio de Janeiro, Rio de Janeiro 20271-110, Brazil; milena.pinto@cefet-rj.br

**Keywords:** 3D inspection, coverage path planning, point cloud analysis, optimization, UAV

## Abstract

Different practical applications have emerged in the last few years, requiring periodic and detailed inspections to verify possible structural changes. Inspections using Unmanned Aerial Vehicles (UAVs) should minimize flight time due to battery time restrictions and identify the terrain’s topographic features. In this sense, Coverage Path Planning (CPP) aims at finding the best path to coverage of a determined area respecting the operation’s restrictions. Photometric information from the terrain is used to create routes or even refine paths already created. Therefore, this research’s main contribution is developing a methodology that uses a metaheuristic algorithm based on point cloud data to inspect slope and dams structures. The technique was applied in a simulated and real scenario to verify its effectiveness. The results showed an increasing 3D reconstructions’ quality observing optimizing photometric and mission time criteria.

## 1. Introduction

Over the last few years, different practical applications emerged requiring periodic inspections to verify possible structural changes, guaranteeing safety through preventive assessment. For instance, large structures, such as dams and slopes, need constant monitoring, and due to the size of these structures, manual inspections are time-consuming and may present risks to humans. In this context, Unmanned Aerial Vehicles (UAVs) arose as a prominent solution to automate this process in a cost-effectively way. In addition, UAVs have positioned at the forefront of different application fields, such as infrastructure inspection [1,2], search and rescue [3,4], delivery [5,6], among others. In the scenario of large structures’ inspections, the UAV may help decrease the mission’s complexity, such as data gathering and geometry land specifications due to its maneuvering flexibility, high versatility, and the possibility of attaching new technologies into it [2].

Inspections on large structures with UAVs should minimize flight time due to battery time restrictions and identify the terrain’s topographic features. In this sense, Coverage Path Planning (CPP) aims at finding the best path to coverage of a determined area respecting the operation’s restrictions [7]. Thus, the development of several algorithms allowed the application in these kinds of processes [8,9,10]. For example, there are several applications, such as underwater inspection [11] and aerospace [12] tasks.

Note that the accurate 3D models are the desired result for inspections. However, some issues remain unsolved from a computational geometry perspective. Path planning refers to finding an optimal route of a moving object from an initial to a final point [13]. Several works have been proposed in the last few years to improve this technique and apply it in the robotics context. In [14], the authors developed a cell decomposition algorithm for robots with contact sensors for covering unknown environments in an online manner. The work of [15] proposed a hybrid methodology for mobile robots on an autonomous mission involving an offline approach that uses the Direct-DRRT* algorithm and the Artificial Potential Fields (APF) algorithm for the online planner. In [16], the authors used a bat algorithm for solving the mobile robots’ global localization problem.

Robots need to perceive the world to work in unstructured environments. In this sense, with the advent of low-cost 3D sensing hardware and in cloud processing, 3D perception in robotics has gained more attraction [17]. Therefore, dense point cloud generation has faced a great advance in the last few years thanks to the rapid development of technologies and algorithms. For instance, Ref. [18] developed an improved delineation method using high-density LiDAR. In [19], the authors worked with image-based 3D reconstruction. They presented a quantitative comparison of several multi-view stereo reconstruction algorithms. The work of [20] proposed an optimization-based algorithm for planar patches extraction from noisy point-cloud data.

3D objects can provide information for creating routes or even for refining previously created paths. It is possible to determine many properties of 3D data objects. However, point cloud data are very unorganized, noisy, and sparse, demanding processing stages to use this information [21]. In addition, in some situations, the foreground is very mixed with the background due to sensor limitations to precisely acquire 3D data. In this sense, point cloud learning has increasing attention with applications in many domains, such as computer vision, autonomous driving, and robotics. The representation of 3D data is generally in different formats, including depth images, point clouds, and meshes. As a commonly used format, point cloud representation preserves the original geometric information in 3D space, representing the environment without discretization [21]. 3D point cloud data are applied for two major applications, including 3D model reconstruction and geometry quality inspection, to represent structures in search of deformations or models [22]. One of the objectives for using point clouds is the temporal comparison looking for deformations in structures in reconstructions performed in different periods. In order to compare two structures, both reconstructions must have points of interest in common [23].

Other recent literature methods include techniques, such as [24,25] that apply GA to obtain shortest path solutions in 3D space. In [26], a GA is used to find viable paths considering radio signal intensity. However, these methods do not include any optimization in terms of image and inspection quality. Other similar methods have also been developed in the last few years for deployed 3D environments, such as Rapidly-exploring Random Tree [27] and Visibility Graph [28]. Still, none of these focused on the specific application shown here. A comprehensive review of similar methods can be found in [29].

Therefore, this research’s main contribution is developing an optimization-based point cloud methodology for application in inspection tasks of large and complex structures, such as slope and dams, which require periodic inspections to verify structural changes. The technique was applied in a simulated and real scenario to show its effectiveness. The other contributions of this research work can be summarized as follows:

- Coverage Path Planning algorithm that optimizes photometric characteristics such as intersection, intersection and incidence angle and UAV flight time using metaheuristics;
- Path change algorithm to increase the quality of points of interest for coupling 3D reconstructions;

The rest of this article is organized as follows. Section 2 shows the proposed optimization-based point cloud methodology and the necessary mathematical foundations for developing it in a real environment. Section 3 presents the results and discussions. The final concluding remarks and ideas for future works are in Section 4.

## 2. Proposed Framework

CPP is the task of determining a path that passes through all points of a determined area. Choset et al. [30] proposed the classification of coverage algorithms in two types: online and offline. The Offline algorithms depend only on stationary information, and the environment is known. On the other hand, online algorithms do not assume complete prior knowledge of the environment to be covered. In addition, they use real-time sensor measurements to scan the target space.

In large structures, such as slopes and hydroelectric dams, UAV missions require extensive pilot experience to cover the large area. In addition, the missions’ repeatability is affected, preventing reconstructions at different times from being compared. Another aspect is the waste of the UAV energy since, in a manual flight, the flight time and the images’ overlay depend only on the operator’s skill. It is possible to generate a mission with optimized aspects concerning the aircraft and the obtained images using information from a previously performed 3D reconstruction. Thus, this research lists the challenges of working with these structures and the missions’ generation. The methods can be divided into the structure’s analysis to be delivered, data filtering, metaheuristic optimization, and dynamic objectives identification.

**Figure 1:**
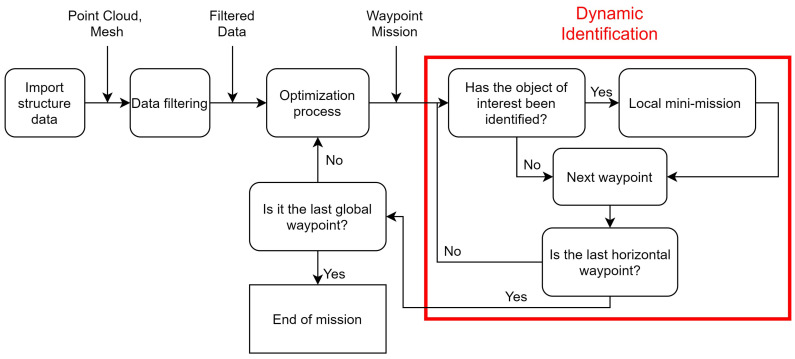
Framework diagram.

Figure 1 presents the framework diagram. Initially, the structures’ data can be imported in the formats of a point cloud, mesh, or structure representing the surface. The data must then be filtered to identify the surface shape, remove outlier, and reduce the optimization algorithm’s points. The optimization algorithm will create a waypoint mission that meets the mission time and photometry criteria. This mission will be sent to the UAV to start the flight. During the route, each waypoint identifies the Point of Interest (POI). In case this point is identified, the framework realizes a local mini-mission, increasing the image data of that region, and then, the UAV goes to the next waypoint. In a negative case, the aircraft will move to the next waypoint. Differently, if the waypoint is the last of the horizontal movement, the UAV checks if it is the global mission’s end. If not, the mission is optimized again to lessen the impacts of local missions and any flight delays. If it is the endpoint, the mission will end, making the UAV returning to the takeoff location. At each waypoint within the horizontal transfer, the framework performs a POI identification.

According to [31], 3D reconstructions can be broadly categorized into three categories: (i) voxel-based representations; (ii) point-based representations; and (iii) mesh representations. Voxel representations are a straightforward generalization of pixels to the 3D case. However, the cost of memory in this representation grows cubically with resolution. In this way, alternative representations are point clouds and meshes. They use appropriate functions to decrease losses without dramatically increasing the cost of memory. However, the point clouds do not have the mesh connectivity structure, and, therefore, this representation needs additional processing steps to extract the geometry from the 3D model [32].

The framework proposed in this article requires the surface’s shape, which can be in any of these categories. The reconstructions must conform to the UAV’s coordinate system, with emphasis on the ECEF and GPS. ECEF, which is an acronym for earth-centered, earth-fixed, is a geographic and Cartesian coordinate system. It represents positions as X, Y, and Z coordinates. The point (0, 0, 0) is defined as the center of mass of Earth [33]. The GPS is a satellite-based radio navigation system representing the terrestrial globe’s position by latitude, longitude, and height relative to an ellipsoidal Earth model [34]. Both position models’ representation can be converted to each other using nonlinear optimization [35]. With the reconstructions in the proper format, it is necessary to filter the points to carry out missions appropriate to these structures.

### 2.1. Data Filtering

The data of the 3D reconstructions are saved in formats unique to these structures, highlighting the Wavefront (.obj), Polygon File Format (.ply), or COLLADA (.dae). These formats present the data, with their peculiarities, in the following form: x position, y position, z position, normal vector [nx, ny, nz], and color [r,g,b] of each point. The positions are the representation—in our case, in ECEF or GPS, of the position of the point.

**Figure 2:**
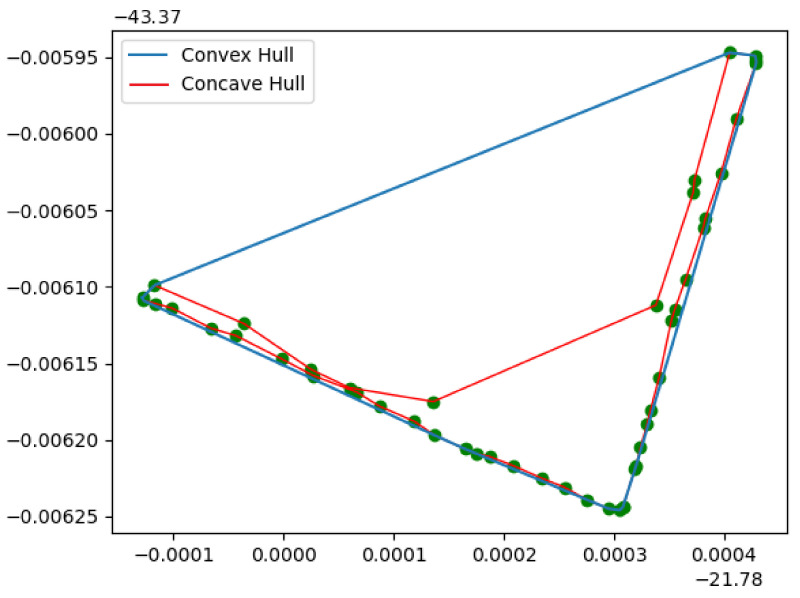
Comparison between Convave Hull and Convex Hull algorithms.

Note the necessity of these points to be filtered to create a mission with this data as a reference, removing outliers and decreasing the optimization algorithms’ points. Some algorithms for this task are the Convex Hull and Concave Hull. The convex hull of geometric objects is the smallest convex set that contains the objects. There are algorithms for points in the literature in two, three, and even Euclidean spaces of higher dimension [36]. The concave hull is an algorithm that finds a concave object that surrounds all points, using methods such as Alpha Shapes or K-nearest neighbor algorithms [37]. Figure 2 shows both algorithms at points in a reconstruction, looking at only one height of the entire reconstruction. The points are green, with the blue line formed by the convex hull and the concave hull’s red line. Although the concave hull has better results in representing the surface, the algorithm’s computational time is higher than the convex hull.

### 2.2. Optimization Process

**Figure 3:**
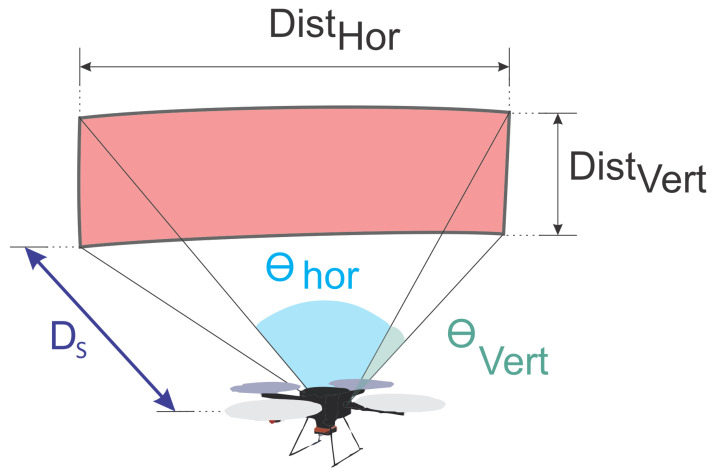
Problem variables.

After analyzing the filters’ points, we have a surface layer that presents the surface information. In this step, the optimization of path planning begins. For photometric issues, the framework considers the intersection among images: the intersection angle and incidence angle. For 3D reconstructions, the number of overlapping photos is an essential factor for these points’ accuracy [38]. Figure 3 illustrates the variables considered in the problem model. The rectangle formed by $Dist_{Vert}$ and $Dist_{Hor}$ forms the Field of View (FOV) of the UAV camera. These dimensions, as seen in Equations (1) and (2), depend on the distance from the UAV to the surface ($D_{S}$) and the horizontal and vertical opening angles of the camera (respectively $\theta_{hor}$ and $\theta_{vert}$):

(1) $$tan\left(\frac{\theta_{hor}}{2}\right)=\frac{Dist_{hor}}{2D_{S}}$$

(2) $$tan\left(\frac{\theta_{vert}}{2}\right)=\frac{Dist_{vert}}{2D_{S}}$$

**Figure 4:**
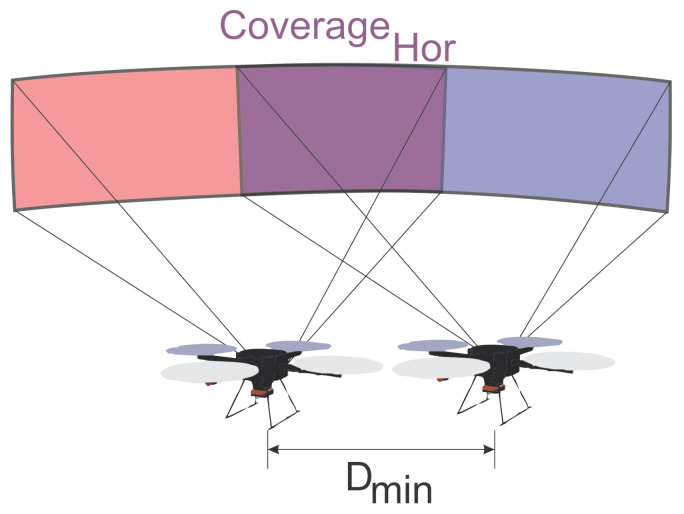
Horizontal coverage.

The mission carried out by the aircraft has the format of horizontal transfers at different heights, and the images will be taken after a $D_{min}$ shift. These displacements create a region of intersection among the photos, as highlighted in color blue in Figure 4. Equation (3) represents the percentage of coverage calculation in relation to $Dist_{Hor}$. The $D_{min}$ distance was designed to capture images with the UAV hovering to prevent the image from losing quality. Suppose the camera can capture a moving image without loss of quality. In that case, the parameter $D_{min}$ will be used to reconstruct the surface shape that will be inspected. It is not necessarily part of the optimization. The image can be captured at a shorter possible distance, and, in Equation (7), the $Time_{shot}$, which is the downtime for image capture, can be zero:

(3) $$Coverage_{hor}=\left(1-\frac{D_{min}}{Dist_{hor}}\right)*100$$

**Figure 5:**
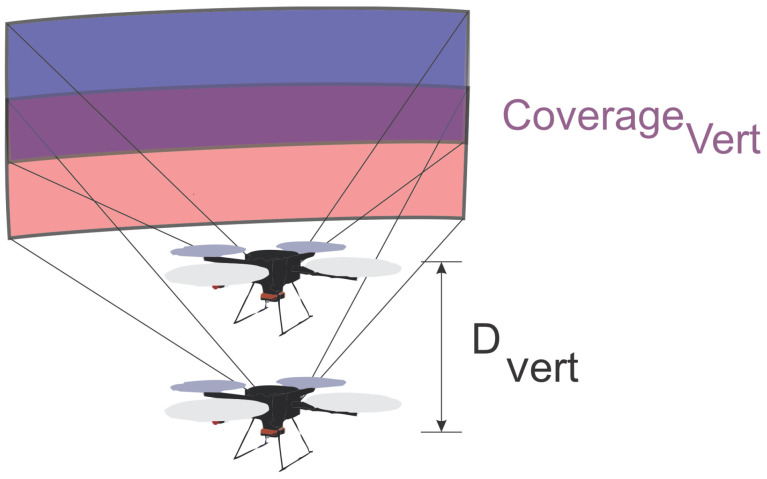
Vertical coverage.

The differences among the missions’ heights also generate a vertical intersection between the photos. The vertical offset ($D_{vert}$) depends on the height of the surface, defined by the difference between the maximum height ($h_{max}$) and minimum height ($h_{min}$), and the number of vertical waypoints ($N_{Vert}$) that have been defined for the mission. Figure 5 illustrates the vertical intersection between photos. Equation (4) shows the calculation of vertical displacement, while Equation (5) presents the vertical coverage:

(4) $$D_{vert}=\frac{|h_{max}-h_{min}|}{\left(N_{Vert}+1\right)}$$

(5) $$Coverage_{vert}=\left(1-\frac{D_{vert}}{Dist_{vert}}\right)*100$$

**Figure 6:**
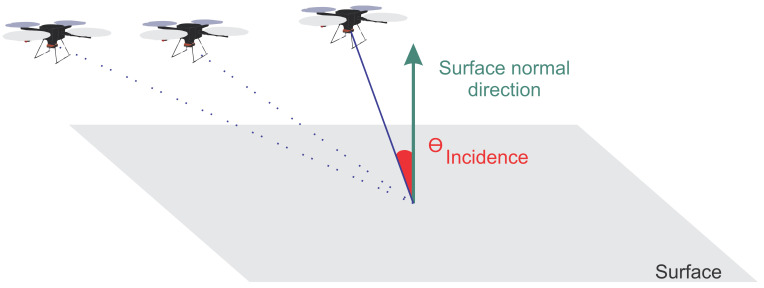
Intersection and incidence angles.

The intersection angle is defined as the angle that surrounds all images taken from that point. When the intersection angle increases, the correspondences may become discontinuous. Note that large intersection angles make image matching difficult, whereas small ones result in low intersection precision [39]. Thus, for better accuracy, it is given that the intersection angle should be close to 90º [38]. The incidence angle is defined as the angle between the image normal and the surface normal. As can be noticed, when it is closer to 0 degrees, the quality of the images is better and thus also the accuracy of the points [38]. Figure 6 shows the angles of incidence and intersection. The blue region represents the intersection region, where the 4 UAVs that can capture the same point are identified. The incidence angle is identified in red, between the UAV and the surface normal.

The objectives considered are to decrease the mission time and increase the intersection area between the images, besides adjusting the angles. For these purposes, the variables are $D_{min}$, $D_{S}$, and $N_{Vert}$. The time is computed considering the distance displaced by the aircraft and its average speed, adding an image capture time at each waypoint, as shown in Equation (7). The problem has multiple objectives, being described through the sum of two factors: (i) Time, and (ii) Photometric fitness, as shown in Equation (6). Time fitness, as shown in Equation (8), is a function that tends to decrease mission time, with a maximum value of fitness equal to 10. The parameter $vel_{UAV}$ is the average speed of the UAV during the mission and $N_{Waypoints}$ is the total number of waypoints in the mission. $D_{T}$ is the distance traveled in each horizontal transfer, calculated through the distances between the points:

(6) $$Fitness=G_{Time}*Fitness_{Time}+G_{Photometric}*Fitness_{Photometric}$$

Many optimization problems involve several objectives that require simultaneous optimization. The difficulty in solving multi-objective problems (MOPs) is that these objectives are often contradictory to each other, which means that an improvement in one of the objectives implies the degradation of one or more of the remaining objectives. In time fitness and photometric fitness, the image objectives tend to create missions with closer waypoints and a greater number of flight layers, increasing the total mission time. There is no single ideal solution for such a situation instead of a set of optimal compensation solutions known as Pareto optimal solutions, called Pareto-optimal Front [40]. Several methods are present in the literature to explore the solutions present in the Pareto set.

A prominent example is the use of scalarization functions, a way of combining multiple objectives into a scalar function, optimizing which will produce one solution to the original MOP [41], this being the method used in Equation (6) to highlight the importance of each of the objectives. Gain $G_{Time}$ and $G_{Photometric}$ control the importance between the time objective and the photometric objective. In the case studied, both values are unitary so that both objectives are explored. Other areas of interest that can be highlighted are Evolutionary Multi-objective Optimization (EMO) [42,43] and Multi-Criteria Decision Making (MCDM) [44,45]:

(7) $$T_{mission}=\frac{D_{T}*N_{Vert}+\left(\right|h_{max}-h_{min}\left|\right)}{vel_{UAV}}+T_{shot}\left(N_{Waypoints}\right)$$

(8) $$Fitness_{Time}=\left(-{\left(T_{mission}\right)}^{2}/20\right)+10$$

The photometric fitness goals are to increase coverage (i.e., horizontal and vertical) and improve the characteristics of the intersection and incidence angles. This objective is represented by a sum of the coverages’ fitness multiplied by the intersection angle gain, as shown in Equation (9). Coverage fitness, as shown in Equation (10), tends to increase coverage, with a maximum value of 5 used for horizontal and vertical coverage. The choice for double maximum value in time fitness highlights this objective concerning photometric, being closely linked to mission security. The values 10 and 5 were chosen empirically:

(9) $$Fitness_{Photometric}=G_{Intersection}*\left(Fitness_{Coverage-Hor}+Fitness_{Coverage-Vert}\right)$$

(10) $$Fitness_{Coverage}=\left(\left(-{\left(Coverage_{\%}-100\right)}^{2}/1000\right)+10\right)/2$$

**Figure 7:**
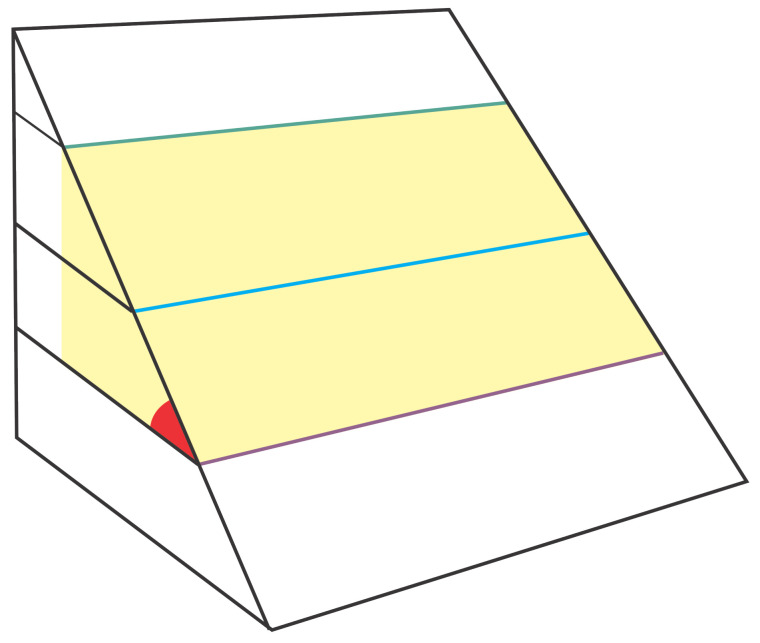
Incidence angle calculation.

The incidence angle can be calculated from three ranges and points from the point cloud. Figure 7 shows the three tracks with different heights. The yellow region is the surface with the points highlighted in green, blue, and purple as the captured points. The incidence angle must be the same as the red angle. In this way, the incidence angle will always be close to 0 degrees, added to the camera’s gimbal angle.

The intersection angle is the difference, in angles, of two previous and two posterior positions of the UAV ($Dif_{Ang}$). A gain is added to the cover fitness to identify how far it is from the 90-degree angle. Equation (11) shows the Gaussian function of the intersection gain. The goal is a Gaussian that ranges from 60 to 120 degrees, with a unit value of 90 degrees:

(11) $$G_{Intersection}=e^{\frac{-{\left(Dif_{Ang}-90\right)}^{2}}{450}}$$

**Figure 8:**
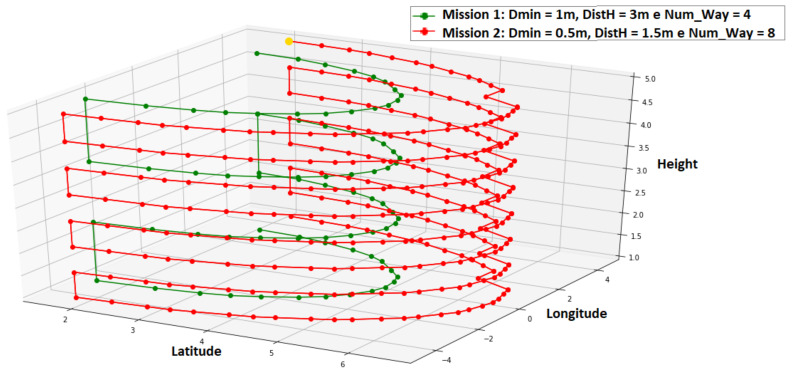
Mission example.

This variable’s purpose is to create missions like those shown in Figure 8. The green mission has $D_{min}$ = 1 m, $D_{S}$ = 3 m, $N_{Vert}$ = 4, while the red mission has $D_{min}$ = 0.5 m, $D_{S}$ = 1.5 m, $N_{Vert}$ = 8. It is noticed that the mission in green is more distant from the surface ($D_{S}$ greater), having less points of image capture because of greater $D_{min}$. The number of horizontal bands is also reduced by the smaller number of vertical waypoints.

With this fitness configuration, the search for better parameters can use any metaheuristic algorithm. For instance, Genetic Algorithm (GA) [46], Particle Swarm Optimization (PSO) [47], Bat Algorithm (BA) [48], Ant Colony (AC) [49], or other methods can be used in the optimization.

Table 1 summarizes the constants with their equivalent units. $N_{Vert}\in N$ and $N_{Waypoints}\in N$ represent numerical values for the number of vertical waypoints and the total of waypoints, respectively.

### 2.3. Dynamic Identification

For the coupling of 3D reconstructions and analysis of them, some landmarks must have great prominence on the surface. These regions need closer flights to create a dense cloud of top-quality points. If the entire mission uses this approach, the UAV’s flight time will last a long time, and the possibility of covering large surfaces will be limited. Thus, a mini local mission was thought of when an object of interest was identified. For identifying objects, the most various algorithms can be used, some using OPENCV [50,51].

**Figure 9:**
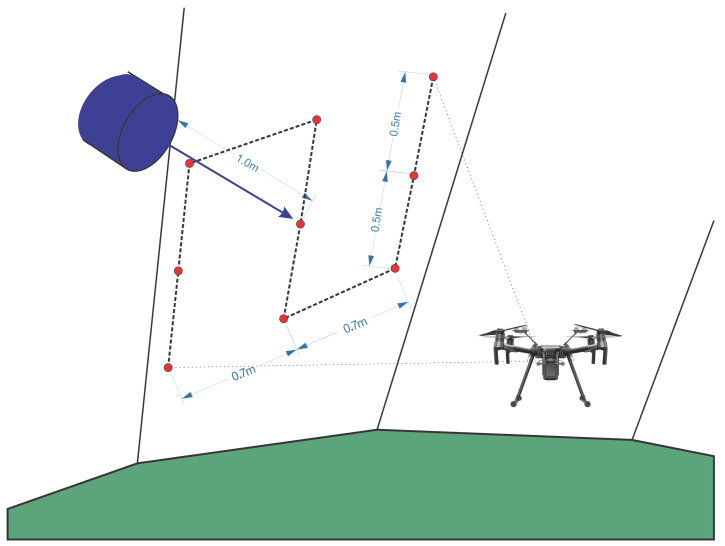
Mini-mission.

After the object is identified, it is necessary to create a local mini-mission. This mini-mission consists of an approximation of one meter from the surface, using a camera or proximity sensors. Nine points are made in a vertical mission, ranging from 0.5 m from the UAV’s current height and 0.7 m in the horizontal direction. After completing the task, the UAV returns to the waypoint and continues its mission. Figure 9 shows an example of the local mini-mission when a blue object is identified on the surface.

Therefore, the flight plan is divided into several horizontal planes. At the end of each horizontal plan, optimization is performed again, considering the remaining flight time and the space to be surveyed. The remaining time is calculated using the maximum mission time minus the time elapsed until the end of the horizontal plane. On the other hand, the remaining area is the horizontal plane’s height below the drone to a height of one meter from the ground. The objective of performing the optimization again is to allow the algorithm to adapt to the dynamic identification missions and possible losses during the flight.

Algorithm 1 demonstrates the decision process of the presented methodology. DATA, filtered_DATA, $T_{Mission}$, $T_{Max}$ are the surface data, the filtered data, the available mission time, and the maximum mission time, respectively. The support variables $Mission_{End}$ and $Horizontal_{End}$ represent whether the mission was completed globally or in horizontal expeditions. The filters, Optimizer, and Identification_function functions represent the filtering of the surface data, optimizing missions based on metaheuristics, and identifying objects of interest.
**Algorithm 1:** Decision process of the proposed methodology.
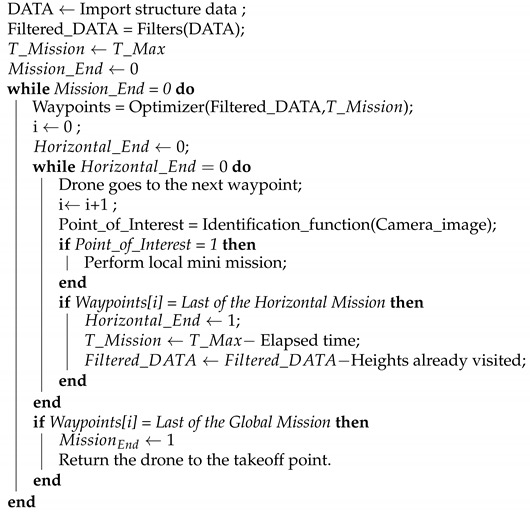


## 3. Results and Discussion

**Figure 10:**
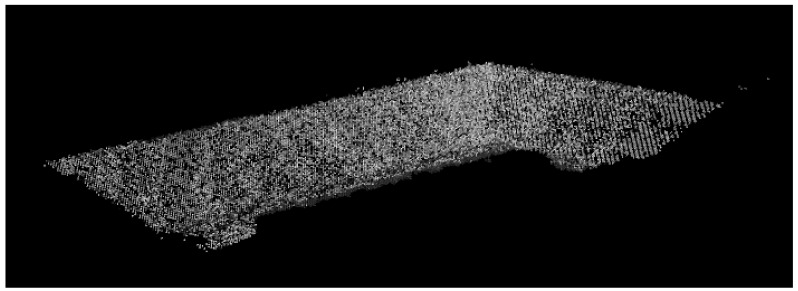
Reconstruction performed through the manual flight.

A manual flight was performed for 3D reconstruction on a slope located at the Federal University of Juiz de Fora, Brazil, to test the proposed framework. The objective is to perform a 3D reconstruction of this structure, as shown in Figure 10. The reconstruction was based on 300 images. Two test environments were used to validate the framework, a simulation environment in the Gazebo-ROS [52], and a real surface itself.

**Figure 11:**
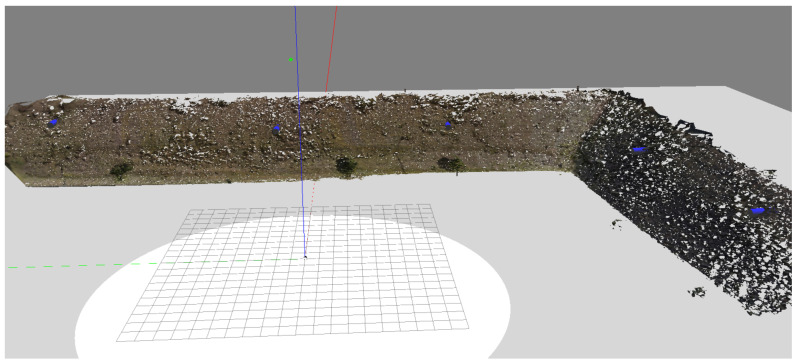
Gazebo World.

**Figure 12:**
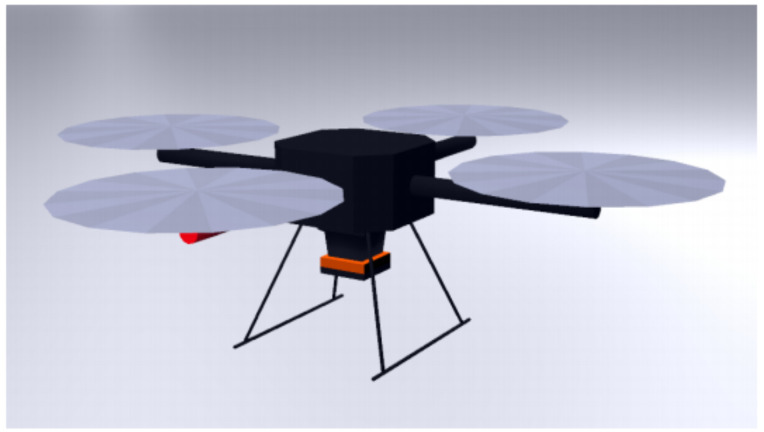
Hector_Quadrotor Model in Gazebo-ROS.

Note in Figure 11 that a world was created in Gazebo-ROS with the presence of the slope and UAV “Hector_Quadrotor” [53]. The model in the UAV Gazebo is in Figure 12.

The chosen metaheuristic optimization methods are the Genetic Algorithm and Bat Algorithm. Fifty initial random populations were created and made available for each algorithm with their respective number of individuals to compare each methodology. The initial population was created using random values that vary according to Table 2. A new set is created for each iteration of the metaheuristic. Still, the same population is used in the GA and in the BA to prevent the initial population’s characteristics from changing the algorithms’ performance.

The version of the GA used is the version for real numbers was proposed by Michalewicz et al. [54]. The mutation and recombination operators are non-uniform mutation and arithmetic crossover, respectively. The selection method is the roulette wheel. Other operators have been tested. These were the ones that obtained the best results. The BA was used in its original version proposed by Yang [48].

**Figure 13:**
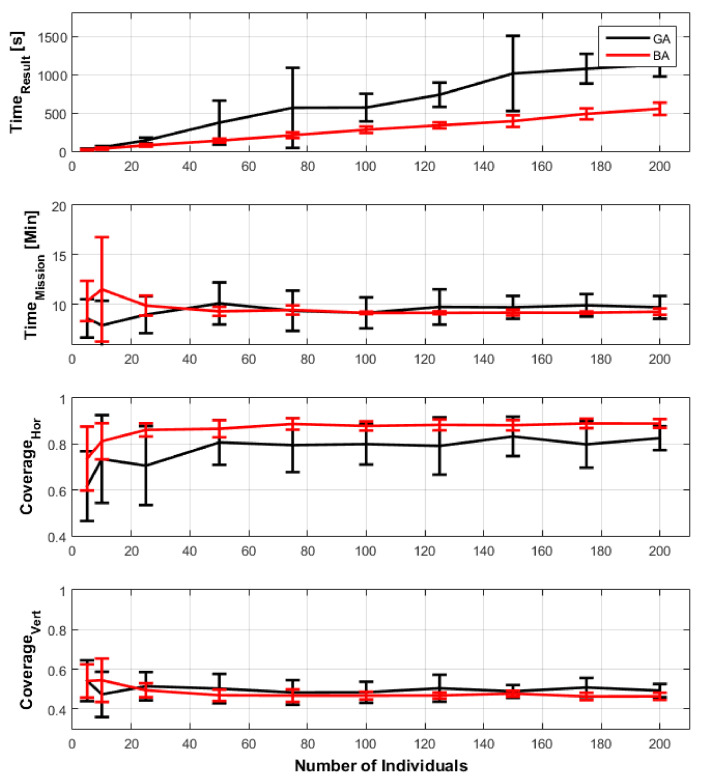
Comparison between GA and BA and its impact comparing numbers of individuals in the population with the time to find the results and objectives of the meta heuristics: time of mission, horizontal, and vertical coverage.

The objective is to analyze each algorithm’s behavior while the variation in the number of individuals, considering average, maximum, and minimum values. The number of epochs available is 100, where this is the stopping criterion. Figure 13 presents the comparison in the following criteria:

1. Time to get the answer ($Time_{Result}$);
2. Time of mission ($Time_{Mission}$);
3. Vertical coverage;
4. Horizontal coverage.

Regarding finding a solution, the BA presented a better response in all population variations, with lower averages than the GA. In addition, the deviations were smaller, being recommended to avoid wasting time during the flight. In the first result regarding the mission time that the aircraft performs, the genetic algorithm presented better results with a population of 5 and 10 individuals. However, with the increase in population, BA had an average closer to that found by GA, but with fewer deviations. In the result of horizontal coverage, the BA had a better result at all levels, with less variation.

Moreover, finally, regarding vertical coverage, the results were very close. Thus, analyzing the data in Figure 13, the BA had a better performance than the GA. Note that the bat algorithm with a population of 50 individuals was chosen to avoid large variations in the results without affecting the increase in computational cost and time to acquire them.

The UAV’s average speed should be chosen to cover the entire surface, avoiding waste to the equipment. A high speed makes the UAV reach the point faster and allows more coverage. However, if the waypoints are close, it causes losses with the high deceleration to capture the image. It may even be necessary for the drone to return to the position if it exceeds the image to capture the waypoint’s position. Low speeds tend to be safer to allow the mission to stop if there is a problem with the equipment, but the potential for surface coverage is reduced. Speeds between 0.1 m/s and 3 m/s were tested. Our missions were around 200 m long, speeds around 0.3 m/s were chosen so that, together with the aircraft’s image capture and rotation, the total mission time was less than the maximum battery.

**Figure 14:**
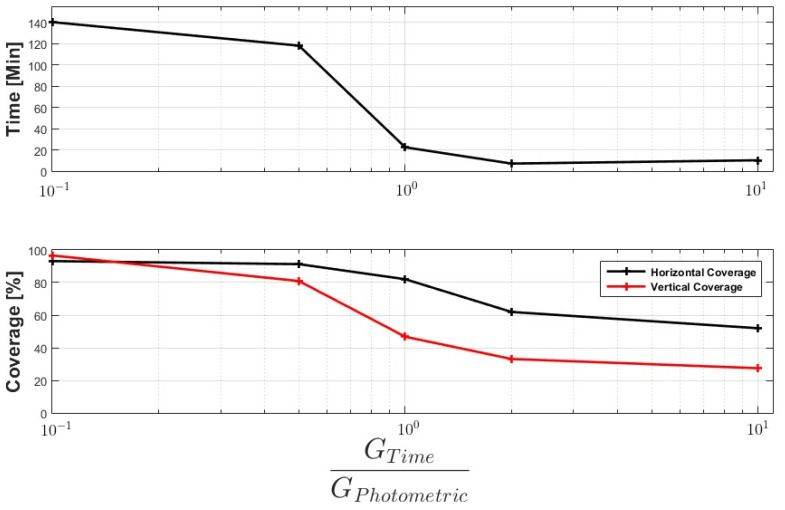
Comparison between $G_{Time}$ and $G_{Photometric}$ and its impact in objectives of the meta heuristics: time of mission, horizontal, and vertical coverage.

In order to verify the impact of the $G_{time}$ and $G_{Photometric}$ gains on the proposed objectives, we create some scenarios to simulate the ratio with the gains, $\frac{G_{time}}{G_{Photometric}}$, varying between [0.1 and 10]. The simulation creates 50 populations for each ratio between the gains, and the result presented is the average among the answers. Figure 14 shows the result. When the ratio favors the photometric objective, the mission time goes to values close to 150 min, requiring at least eight flights with 20 min (battery safety time). When the relationship tends towards time, the mission manages to behave in a single flight, approximately eight minutes. The main factor controlled by the relation is $N_{Vert}$, which directly relates to vertical coverage. Missions with robust photometric criteria had $N_{Vert}$ varying between 9 and 15, while in missions with time as the main factor, $N_{Vert}$ varied between 3 and 4.

Flights were performed with different distances to understand the impact of distance to surface on reconstruction. There were five flights for each distance; the result is the average. Table 3 shows the results for missions with and without the presence of local missions. It is noticed that, for flights without local missions, the closer to the surface, the more points were created in 3D reconstruction. However, missions with this short distance and causing security problems with GPS errors significantly increase the mission time. Thus, it was proposed to use local missions to increase density in some specific points. Vegetation on the surface was chosen as a point of interest. In the reconstruction with three meters of distance, the point density increase was around 51%, while, in the 5 m, it was 54%. The increase in time to perform the missions was 10% to 20% of the time spent.

The same study was carried out for the number of waypoints, for missions with a distance of two meters from the surface of five meters high and a 60º camera opening. Table 4 shows the results. Note that the greater number of waypoints increases the density of points in 3D reconstruction, justifying the increase in vertical coverage being one factor of the fitness function. However, the increase of $Num_{W}ay$ drastically increases the mission time. The number of optimizations made at the end of each horizontal transfer generates missions with much longer times without a significant density increase. The care that should be taken is to avoid minimal transfers, as $Num_{W}ay$ is equal to 2, where the drop in density was significant.

In relation to the parameter $D_{min}$, we have the results presented in Table 5. The results were made with $D_{S}$ = 2 m, $N_{Vert}$ = 6 and horizontal opening of the camera of 100º. The value is strongly linked to the number of points in the 3D reconstruction. Note that the greater the horizontal coverage, the greater is the density of points. Suppose the camera can capture the image without stopping the UAV. It is recommended to increase the maximum number of images possible since increasing the number of points with the capture stop increases the mission time.

With the results presented in Table 3, Table 4 and Table 5, it is possible to note that, for the production of an adequate mission, the UAV must make more horizontal transfers, as close as possible to the surface. Besides, points of image capture close. However, this photometric objective to 3D reconstruction increases the mission time, as the UAV will not cover the entire target surface in one mission. In this way, metaheuristic algorithms balance these objectives, enabling a 3D reconstruction with quality and adapting the whole surface’s mission time. Another detail is the possibility of meeting the distance objective to the surface in some critical locations of the task, increasing the quality of 3D reconstruction in POI to the operator. In this way, the mission will have a greater distance on the surface in general and, in small parts, will have an approach to the surface.

**Figure 15:**
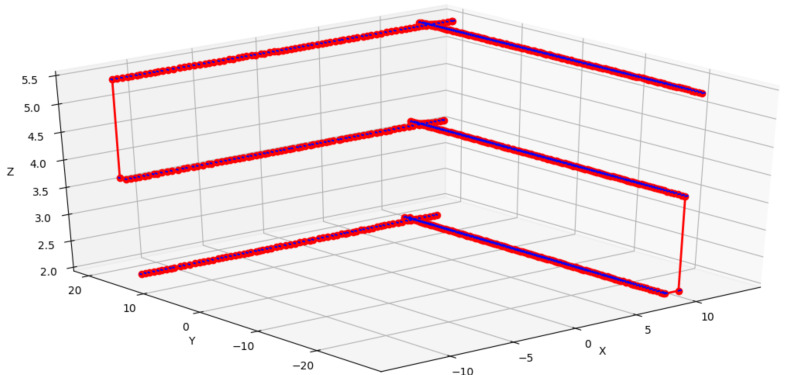
Initial Mission in Gazebo-ROS.

**Figure 16:**
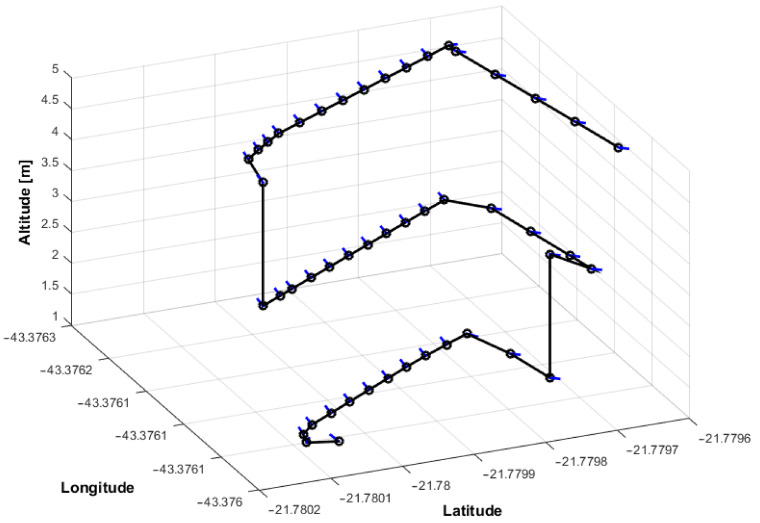
Initial Mission in the real-world.

Table 6 shows the results of the mission in the Gazebo-ROS simulator. This mission consisted of three horizontal missions, the first planned in Figure 15. After each transfer, the next optimizations were performed, with the results of stages 2 and 3. It is noticed that the distance to the Slope remained with small variation, and the same occurs with the distance among the horizontal waypoints. The first stage chose a mission with three vertical waypoints between 1 and 5 m (i.e., slope height). The second was 2 points between 1 and 3.5 m. Finally, the third was a mission at an altitude of 1.5 m. The total mission time was 13 min, less than the 15 min planned for the task. The chosen speed was 0.3 m/s. The aircraft traveled the entire course of the structure in less time than was available for it. The images were taken with the UAV hovering for two seconds to capture images to avoid deformations in the images. Initially, the flight was performed with 3D reconstruction in a simulated environment to improve the mission’s safety, avoiding finding results that did not meet the maximum flight time. After the simulation, the mission was relocated to the real environment. Another feature that had to be implemented is the conversion of distances to meters, as seen in Figure 15 for GPS, showed in Figure 16.

**Figure 17:**
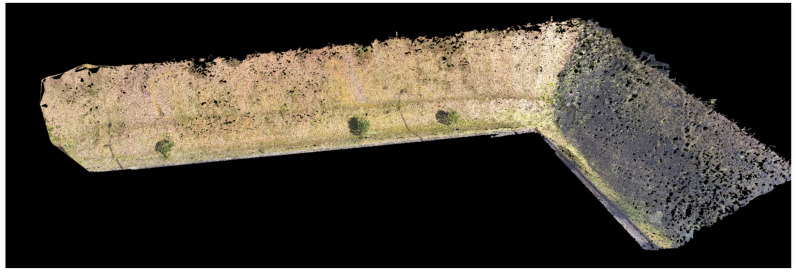
Optimized 3D reconstruction.

Figure 16 presents a mission test performed in a real Slope. The drone used was a DJI Phantom 4 (Nanshan, Shenzhen, China). Figure 17 shows the 3D reconstruction using 80 images. It is possible to conclude that the objective of an optimized autonomous mission for 3D reconstructions has been achieved successfully. It is necessary to increase the number of points at the end of the mission to improve performance, creating images that will only be used in part for 3D Reconstruction.

## 4. Conclusions and Future Work

The proposed research work presented a framework for coverage path planning optimization using a dense point cloud as information from the surface. The main idea is to provide reliable information for periodic inspections in enormous structures to verify possible changes. The data can be used to create routes or even to refine previously created paths. The proposed technique was evaluated in simulation and real scenarios, generating missions with time and photometric optimizations. The results showed good responses to the problem, avoiding wasted energy from the UAV and a specialized operator’s need.

The methodology shows an increase in 3D reconstructions’ density, observing photometric criteria, and equalizing it with the maximum mission time. Note that the objective was achieved, since the increase in the number of vertical transfers and the capture points’ approach significantly increased the number of points in the same region of the 3D reconstruction. Another aspect is the insertion of local approach missions, allowing a sweep in larger areas and increasing density at specific points.

In terms of evaluation, this research opens the possibility of several future works. For example, in addition to more photometric parameters, such as intersection angles among the photos, incidence angles, and features, it will be researched to add the reconstruction’s quality as a parameter of the optimization.

## Figures and Tables

**Table 1:** Summary of constants.

Constant	Explain	Dimension
$D_{min}$	Horizontal Distance between two images	m
$D_{S}$	Distance to Surface	m
$N_{Vert}$	Number of Vertical Waypoints	
$\theta_{hor}$	Opening angle of the camera—Horizontal	º
$\theta_{vert}$	Opening angle of the camera—Vertical	º
$Dist_{hor}$	Field of View—Horizontal	m
$Dist_{Vert}$	Field of View—Vertical	m
$Coverage_{hor}$	Intersection between two images—Horizontal	%
$Coverage_{Vert}$	Intersection between two images—Vertical	%
$D_{vert}$	Vertical displacement between two horizontal transfers.	m
$G_{Time}$	Time Fitness Gain	[0, 10]
$G_{Photometric}$	Photometric Fitness Gain	[0, 10]
$h_{max}$	Maximum surface height	m
$h_{min}$	Minimum surface height	m
$D_{T}$	Distance traveled in each horizontal transfer	m
$N_{Waypoints}$	Total number of mission waypoints	
$vel_{UAV}$	Average UAV speed during the mission.	m/s
$T_{mission}$	Total mission time	s
$G_{Intersection}$	Intersection Angle Gain	[0, 1]
$Dif_{Ang}$	Intersection angle	º
$T_{shot}$	Image capture time	s

**Table 2:** Parameters used to create the initial population.

Variable	Range	Representation
$D_{min}$	[0.1, 20]	R
$D_{S}$	[1, 20]	R
$N_{Vert}$	[1, 10]	N

**Table 3:** Comparison between the variation of $D_{S}$ and its impact on the amount of points of 3D Reconstruction and the Mission Time.

$D_{S}$ [m]	Number of Points	Mission Time [s]	Local Missions
1	133,702	508	No
3	62,438	238	No
5	59,465	179	No
3	94,323	270	Yes
5	91,696	213	Yes

**Table 4:** Comparison between the variation of Num_WaY and its impact on the amount of points of 3D Reconstruction.

$N_{\mathrm{Vert}}$	Number of Points	${\mathrm{Coverage}}_{\mathrm{Vert}}$ [%}
6	133,702	57.4
5	121,654	50.3
4	116,096	40.4
3	103,124	25.5
2	51,980	0.7

**Table 5:** Comparison between the variation of $D_{min}$ and its impact on the amount of points of 3D Reconstruction.

$D_{\min}$	Number of Points	${\mathrm{Coverage}}_{\mathrm{Hor}}$ [%}
0.6	112,257	85
1.2	62,147	70
1.71	48,927	57.1
2	43,781	50
2.4	37,080	40

**Table 6:** Optimization in Gazebo-ROS.

Stage	$D_{\min}$ [m]	$D_{S}$ [m]	$N_{\mathrm{Vert}}$	Mission Time [Min]	Coverage Hor [%]	Coverage Vert [%]
1	1.04	1.71	3	10.59	82.38	47.82
2	0.89	1.96	2	7.40	86.80	49.97
3	1.37	2.05	1	4.60	80.66	46.38
